# Risk Models to Predict Screen-Detected and Interval Breast Cancers in Population Mammography Screening Participants

**DOI:** 10.3390/cancers17050810

**Published:** 2025-02-26

**Authors:** Naomi Noguchi, Armando Teixeira-Pinto, Michael Luke Marinovich, Dominique Claire Louw, Elizabeth Jane Wylie, Nehmat Houssami

**Affiliations:** 1 Sydney School of Public Health, Faculty of Medicine and Health, University of Sydney, Camperdown, NSW 2050, Australia; 2 BreastScreen Western Australia, Perth, WA 6000, Australia; 3 The Daffodil Centre, University of Sydney (a Joint Venture with Cancer Council NSW), Camperdown, NSW 2050, Australia

**Keywords:** breast neoplasms, mammography, mass screening, early detection of cancer, risk factors, interval cancer

## Abstract

Aim: The aim of this study was to determine whether women at risk of having screen-detected (including detected at advanced stage) and interval breast cancer can be accurately identified using conventional risk factors collected by national screening programs. Methods: All 1,026,137 mammography screening examinations for 323,082 women attending the BreastScreen Western Australia program (part of Australia’s national biennial screening program) in July 2007–June 2017 contributed to models for predicting screen-detected breast cancers, screen-detected advanced cancers (≥pT2), and interval cancers. Results: In total, 7024 screen-detected (1551 in situ, 5472 invasive, of which 1329 were ≥pT2) and 1866 interval cancers (76 in situ, 1790 invasive) were diagnosed. In a multivariable model for screen-detected cancers, the ORs for the oldest age groups were 2.56 (CI 2.32–2.82) for 60–69 years and 3.60 (CI 3.23–4.00) for ≥70 years, and the OR for symptoms was 7.44 (CI 6.76–8.20). These associations were stronger for screen-detected advanced cancers. First-degree family history and a personal history of breast cancer were also associated with risk. In a multivariable model for interval cancers, the HR for dense breasts was 2.36 (CI 2.14–2.61) and the HR for symptoms was 3.27 (CI 2.53–4.24); family history and recent hormone replacement therapy use were also associated with risk. The areas under the receiver operating characteristic curves were 0.643 (CI 0.636–0.650) for screen-detected cancers, 0.651 (CI 0.638–0.664) for screen-detected advanced cancers, and 0.706 (CI 0.690–0.722) for interval cancers. Conclusion: Older age and symptoms were the strongest predictors of overall and advanced screen-detected breast cancers. Dense breasts and symptoms were the strongest predictors of interval cancers. All models had moderate discrimination, approximating that for established models.

## Simple Summary

We developed risk models to predict screening outcomes using conventional breast cancer risk factors that are routinely collected by national screening programs. Older age and symptoms were the strongest predictors of overall and advanced screen-detected breast cancers. Dense breasts and symptoms were the strongest predictors of interval cancers. These models were able to discriminate risk similarly to established models that use a more extensive range of risk factors. This work provides new knowledge to inform discussions of and research plans for risk-stratified (individual risk-based) breast cancer screening in population-based screening programs.

## 1. Introduction

In Australia, the publicly funded BreastScreen program currently provides mammography screening, targeting women aged 50–74 years aiming to decrease breast cancer deaths [1]. BreastScreen Australia is similar to many organized population-based breast cancer screening programs in Europe, Canada, and New Zealand. There has been interest worldwide in risk-stratified screening which aims to tailor screening frequency and/or imaging modality based on an individual’s breast cancer risk [2].

Currently, the BreastScreen Australia program collects information from participants on conventional breast cancer risk factors such as personal and first-degree family history of breast cancer, history of biopsy for benign breast disease, hormone replacement therapy, and symptoms that are suggestive of breast cancer. The program does not collect more extensive family history, genetic, hormonal/reproductive, and lifestyle factors. Population-based breast cancer screening programs in other countries generally collect similar datasets.

To inform the discussion of risk-stratified screening, we have previously reported the cancer detection rate at screening (CDR) and interval cancer rate (ICR) by available conventional risk factors and age groups [3]. However, it is unclear if it is possible to accurately identify women who are at an increased risk of developing breast cancer using conventional breast cancer risk factors. As an intermediate step, it would be useful to predict risks of screen-detected cancers (including rates of advanced cancers) and interval cancers separately to inform the potential for risk stratification using existing data collections in population breast screening programs.

The primary aim of this study is to assess the discriminatory accuracy of the set of conventional breast cancer risk factors on screening outcomes (CDR, advanced CDR, and ICR separately). This work can inform the potential to use conventional risk factors to discriminate screen-detected and interval cancer risks and will provide new knowledge to inform discussions of or research plans for risk-stratified breast cancer screening in population-based screening programs.

## 2. Materials and Methods

### 2.1. Participants

All mammography screening episodes in women aged ≥ 40 years in the BreastScreen Western Australia (BSWA) program from 1 July 2007 to 30 June 2017 were included in the analyses using routinely collected clinical and administrative data.

The program invited women aged between 50 and 69 until 30 June 2013 and between 50 and 74 from 1 July 2013 to receive free mammograms every 2 years. Women aged between 40 and 49 years were also eligible to attend, though they not actively invited. Women in the target age group who were deemed high-risk due to the following characteristics were invited for annual screening: strong family history (≥2 affected first-degree relatives or ≥1 first-degree relative diagnosed at <50 years or with bilateral disease), personal history of breast and/or ovarian cancer, or previous diagnosis of benign lesions associated with increased risk (atypical ductal hyperplasia and lobular carcinoma in situ). These women are at increased risk of breast cancer (but do not represent the highest risk group that comprises gene mutation carriers), and population screening programs allow women at increased risk to participate. Therefore, the study population was representative of population screening participants.

Participants in the BSWA program provide written consent for their data to be used for research and quality assurance each time they attend a screening. This study was approved by the Governance, Evidence, Knowledge & Outcomes Ethics Committee, Quality Improvement Women’s Health, Genetics & Mental Health, Women and Newborn Health Service (reference number 34263).

### 2.2. Measurement

The BSWA program routinely collects information on demographic characteristics, breast cancer risk factors, and breast symptoms using a self-administered registration form [4]. The outcomes of all screening mammograms and further assessments for recalled individuals are routinely recorded in the Mammographic Screening Registry.

Collected variables included age, postcode, screening round, time since last screen for repeat screens, mammographic breast density, personal history of breast and ovarian cancer, first-degree family history of breast cancer, use of hormone replacement therapy (HRT) in the past 6 months, a history of surgery or biopsy for a benign breast lesion, and self-reported breast symptoms (“breast lump”, “nipple discharge”, or “other concerning symptoms or breast change”). Symptoms are recorded as present or absent and do not specify which symptom each participant presented with. Age was categorized into four groups of 40–49, 50–59, 60–69, and ≥70 years. The Index of Relative Socio-economic Disadvantage (IRSD) was used to determine socio-economic status (SES) based on postcode [5]. Area-based quintiles within Western Australia from the nearest census year were used. Women were considered to have dense breasts if at least one of the two radiologists who double-read the mammogram visually classified it as showing heterogeneously or extremely dense breasts [6]. Breast density was recorded only for women who were not recalled for further testing.

### 2.3. Outcomes

Screen-detected cancers were defined as cancers detected following abnormal screening mammograms and a recall for assessment. Pathological tumor size pT2 and above was considered screen-detected advanced cancer.

Interval cancers were defined as cancers in women whose screen mammograms were negative (i.e., not recalled) and in whom breast cancer diagnosis occurred before the next scheduled screen (two years for most women or one year for those meeting criteria for high risk). If biennial screeners presented before 730 days or annual screeners before 365 days with a symptom and then were diagnosed with a cancer in the same breast, they were considered to have interval cancers.

### 2.4. Statistical Analysis

Characteristics of the cohort were summarized descriptively using percentages for categorical variables.

Models were developed to predict screen-detected cancers, screen-detected advanced cancers, and interval cancers. For each model, univariable logistic regressions for screen-detected cancers and univariable Cox regressions for interval cancers were fitted to determine the relationship between each candidate variable and the outcome. Then, all variables below were forced into the multivariable models, as they were all deemed clinically important. Candidate variables to predict overall and advanced screen-detected cancers included age group, postcode-based SES, time since last screen, personal history of breast cancer, family history of breast cancer, breast density, use of HRT in last 6 months, history of benign biopsy, and breast symptoms. Candidate variables to predict interval cancers included age group, postcode-based SES, time since last screen, family history of breast cancer, breast density from the current screen, use of HRT in last 6 months, history of benign biopsy, and breast symptoms. The area under the receiver operating characteristic curve (AUC-ROC) was determined for each model.

Since breast density was only reported for women who had no abnormality on their current mammogram, the models for total and advanced CDR included breast density derived from the prior 27 months. The time since last screen and breast density from a recent screen were used to form one variable to include information on density in the model. Personal history could not be included in the model for interval cancers because recurrent or second breast cancers are not routinely reported to cancer registries; therefore, we cannot reliably identify interval cancers in that subgroup.

Given that our program collects self-reported symptoms (if any), a sensitivity analysis excluded the symptom variable to report the models for asymptomatic screened populations.

Statistical analysis was performed using R version 4.3.3 [7].

## 3. Results

Between 1 July 2007 and 31 June 2017, 323,082 women aged ≥ 40 years had a total of 1,026,137 screens in BSWA. In total, 7024 screen-detected cancers (1551 in situ, 5472 invasive, of which 1329 were ≥pT2 category) and 1866 interval cancers (76 in situ, 1790 invasive) were diagnosed. Overall, CDR was 68 (95% CI 67–70) per 10,000 screens and ICR was 9.7 (95% CI 9.2–10.1) per 10,000 women-years.

Supplementary Table S1 summarizes the characteristics of women at each screening episode. The mean age at the time of screening was 58.5 (SD +/− 8.6) years (range 40–98). The majority of screening episodes were repeat screens between the 15th and 26th months, and the majority were not associated with any risk factors.

Table 1 shows univariable and multivariable analyses for predicting screen-detected breast cancers.

Modeling the risk of screen-detected cancers showed symptoms had the highest OR (7.44 (CI 6.76–8.20) followed by oldest age groups (OR 2.56 (CI 2.32–2.82) for 60–69, 3.60 (CI 3.23–4.00) for ≥70) in the multivariable analysis. Dense breasts (compared among repeat screens that returned around the 2-year mark (≥15, <27 months), which is the recommended interval for most women), family history, and personal history of breast cancer, HRT use, and history of benign lesions were all associated with a less than 50% increase in the OR in the multivariable model. The AUC-ROC for the model to predict screen-detected cancers was 0.643 (CI 0.636–0.650) (Supplementary Figure S1.1). The Hosmer and Lemeshow goodness of fit had a marginally significant *p*-value of 0.032 due to the large sample size, but the calibration of the model did not show evidence of a lack of calibration (Supplementary Figure S1.2).

The OR for age increased from univariable to multivariable analysis, the OR for time since last screen of <15 months in univariable analysis diminished when adjusted for other risk factors, whilst the OR was unchanged between univariable and multivariable models for most variables. A personal history of ovarian cancer had a lower risk of screen-detected cancer compared to those without a history of ovarian cancer in the univariable model (OR 0.57 (CI 0.36–0.86)).

When restricted to screen-detected advanced cancers, associations for symptoms (OR 22.7 (CI 19.6–26.3)) and age (OR 3.18 (CI 2.55–3.97) for 60–69, 4.70 (CI 3.69–5.99) for ≥70) were enhanced compared to the model for predicting screen-detected cancers overall, and these remained the strongest predictors in the multivariable model. Associations for dense breasts (compared among repeat screens ≥15, <27 months since last screen), personal history of breast cancer, and longer intervals since last screen (≥27 months) with screen-detected advanced cancers were also enhanced compared to the model for screen-detected cancers overall, with increases in the OR of 50% to 100% observed in the multivariable model. Of note, the two least disadvantaged SES categories had 20% lower advanced cancer rates. The AUC-ROC for this model to predict screen-detected advanced cancers was 0.651 (CI 0.638–0.664).

Table 2 shows univariable and multivariable analyses to predict interval cancers. Women with symptoms (HR 3.27 (CI 2.53–4.24) and those with dense breasts (HR 2.36 (CI 2.14–2.61)) had the highest HR. Other risk factors were associated with up to a 50% increased risk of interval cancers in the multivariable model, including the oldest age group. Of note, the least disadvantaged SES category had a higher HR in the multivariable model. The AUC-ROC for the model to predict interval cancers was 0.706 (CI 0.690–0.722) (Supplementary Figure S2.1). The Hosmer and Lemeshow goodness of fit was again marginally significant (*p*-value = 0.057), but the calibration of the model did not show evidence of a lack of calibration (Supplementary Figure S2.2).

In the sensitivity analyses limited to asymptomatic women, ORs and HRs did not change substantially, and the AUC-ROC values were 0.623 (CI 0.616–0.630) for screen-detected cancers overall, 0.646 (CI 0.629–0.663) for screen-detected advanced cancers, and 0.6441 (CI 0.630–0.658) for interval cancers.

## 4. Discussion

This study used > 1 million screening mammograms from a large representative cohort of Australian women participating in screening to build statistical models that predict an increased risk of screen-detected breast cancers, screen-detected advanced breast cancers, and interval breast cancers to inform potential use in risk-stratified screening. Presenting to screening with self-reported symptoms was strongly associated with the risk of both screen-detected cancers (seven times the risk compared to asymptomatic screening participants) and interval cancers (three times), and the association was stronger for screen-detected advanced cancers (23 times). Older age was a strong predictor of screen-detected cancers overall (four times for 70 and over) but had a weaker association with interval cancers (40% increase for 70 and over). Dense breasts had a moderate association with screen-detected cancers (40% increase) but a stronger association with interval cancers (twice the risk). Other risk factors had up to a 50% higher risk of overall and advanced screen-detected and interval cancers, except for a lack of association between a history of biopsy showing benign lesions and screen-detected advanced cancers. Specific associations included a first-degree family history of breast cancer (approximate 30% increased risk of screen-detected and screen-detected advanced cancer) and personal history of breast cancer (40% increased risk of screen-detected cancer and 60% increased risk of screen-detected advanced cancer).

Most previous studies that determined risk factors for breast cancer in screening populations predicted overall breast cancer as an outcome instead of screen-detected and interval cancers separately [8], and a previous study that predicted screen-detected and interval cancers separately did not include dense breasts, which is an important risk factor [9]. We developed separate models for pre-specified outcomes because interventions would be prioritized for those at higher risk of interval or screen-detected advanced breast cancer. The magnitude of the risks shown in our models generally aligns with adjusted risk ratios of these risk factors for overall breast cancer risk determined in previous studies [10]. Of note, the least disadvantaged SES groups in our cohort had a lower risk of screen-detected advanced cancer whilst having a higher risk of interval cancers. This may reflect the difference in healthcare-seeking behavior between different socio-economic groups, as was found in previous studies [11,12,13].

Another aim of this study was to investigate whether it is possible to discriminate breast cancer screening outcomes, including the risk of specific outcomes (e.g., interval cancers) based on factors that are routinely collected by the Australian population-based breast cancer screening program. In this study, AUC-ROCs were between 0.643 and 0.706 for screen-detected cancers overall, screen-detected advanced cancers, and interval cancers. The models that were restricted to asymptomatic women (99% of screening participants in our cohort) had AUC-ROC values in the range between 0.623 and 0.646. Commonly used risk models such as the Tyler-Cuzick model [14], Gail model [15], and the Italian model [16] take into account a wider range of risk factors including multigenerational family history, hormonal and reproductive factors, or lifestyle factors. However, a systematic review on the accuracy of breast cancer risk assessment methods [17] has found that studies reported AUC values between 0.55 and 0.65 for existing risk models, indicating moderate accuracy in predicting incident breast cancer. Although we used a less extensive set of risk factors routinely collected in the BreastScreen program, as described in the methods, the AUC-ROC values were moderate and similar to those reported for models using more extensive risk factors.

The major limitation of this study is that we did not have information about some relevant risk factors such as family history on the paternal side, established high-risk gene mutations, or radiotherapy to the chest. However, these are relatively rare, and women with known cancer-predisposing genes are generally managed in specialized clinics. Another limitation is that we did not have interval cancer data in women with a personal history of breast cancer because second breast cancers are not routinely reported to cancer registries in Australia. Some may consider that including symptoms as a variable in the models may not be relevant; however, in reality, a minority of women undergoing screening declare having a breast symptom (1% in our cohort)—hence, we retained it to keep all (consecutive) screened participants and addressed this issue in the sensitivity analysis. Also, although we had breast density data, density for one of the models (for predicting screen-detected cancers) had to be derived from prior mammograms for a subset of participants. Furthermore, these risk models have not yet been validated externally because our aim was not to develop models that would be used in practice widely. Rather, our interest lies in whether it is possible to discriminate breast cancer risk for the three outcomes defined in the study based on conventional risk factors that are routinely collected by our population-based screening programs. This may help inform program-based validation of the models and may guide strategies directed at those at risk of a specific outcome—for example, based on the interval cancer risk model, a modified screening approach for older women with dense breasts would be needed to manage the risk of interval cancer in the Australian screening population.

## 5. Conclusions

In conclusion, using conventional breast cancer risk factors alone, we developed models for predicting the risk of each screen-detected cancer, screen-detected advanced cancer, and interval cancer, and we showed that the models had moderate discrimination. Individual risk factors had varied impacts on these different screening outcomes, although self-reported symptoms and older age were the strongest predictors of overall and advanced screen-detected cancers. Symptoms and breast density were the strongest predictors of interval cancers. The findings from our study may inform the ongoing discussion in breast screening programs worldwide regarding the potential for risk-stratified screening and planning of future research to generate evidence on the effectiveness of this approach.

## Figures and Tables

**Table 1:** Univariable and multivariable models to predict overall and advanced screen-detected cancers.

		All Screen-Detected Cancers			Advanced * Screen-Detected Cancers		
			Univariable	Multivariable		Univariable	Multivariable
Characteristic	Category	N events	OR (95% CI)	OR (95% CI)	N events	OR (95% CI)	OR (95% CI)
Age group	40–49	650	Reference	Reference	140	Reference	Reference
	50–59	2367	1.23 (1.12–1.34)	1.58 (1.45–1.74)	448	1.08 (0.89–1.31)	1.80 (1.46–2.21)
	60–69	2766	1.71 (1.57–1.87)	2.56 (2.32–2.82)	491	1.41 (1.17–1.70)	3.18 (2.55–3.97)
	≥70	1237	2.65 (2.41–2.92)	3.60 (3.23–4.00)	250	2.47 (2.01–3.05)	4.70 (3.69–5.99)
Postcode-based SES (IRSD)	1st quintile (most disadvantaged)	691	Reference	Reference	150	Reference	Reference
	2nd quintile	1678	1.04 (0.95–1.13)	1.00 (0.91–1.09)	320	0.91 (0.75–1.11)	0.87 (0.71–1.05)
	3rd quintile	1396	1.00 (0.91–1.09)	0.98 (0.90–1.08)	281	0.93 (0.76–1.13)	0.91 (0.74–1.10)
	4th quintile	1066	0.95 (0.86–1.05)	0.96 (0.87–1.05)	186	0.76 (0.62–0.95)	0.77 (0.62–0.96)
	5th quintile (least disadvantaged)	2155	0.93 (0.85–1.01)	0.95 (0.87–1.03)	385	0.76 (0.63–0.93)	0.79 (0.66–0.96)
Screen round/time since last screen and breast density from last screen	First screen	1280	1.63 (1.52–1.75)	2.30 (2.12–2.49)	320	2.65 (2.26–3.11)	3.44 (2.87–4.12)
	<15 months, not dense	262	1.14 (1.00–1.30)	0.89 (0.77–1.02)	34	0.96 (0.66–1.35)	0.74 (0.51–1.07)
	<15 months, dense	80	1.12 (0.89–1.39)	0.98 (0.77–1.23)	14	1.28 (0.71–2.10)	1.18 (0.68–2.04)
	<15 months, no data on density	332	1.76 (1.56–1.97)	1.07 (0.90–1.27)	49	1.68 (1.23–2.26)	0.91 (0.61–1.37)
	≥15, <27 months, not dense	1931	Reference	Reference	296	Reference	Reference
	≥15, <27 months, dense	687	1.24 (1.13–1.35)	1.37 (1.25–1.50)	136	1.60 (1.30–1.95)	1.83 (1.49–2.25)
	≥15, <27 months, no data on density	733	1.12 (1.03–1.22)	1.15 (1.05–1.25)	108	1.08 (0.86–1.34)	1.10 (0.88–1.38)
	≥27 months, no recent data on density	1715	1.67 (1.57–1.79)	1.59 (1.48–1.70)	372	2.36 (2.03–2.75)	2.11 (1.81–2.47)
First-degree family history of breast cancer	Did not report any	5277	Reference	Reference	1015	Reference	Reference
	Yes	1743	1.27 (1.21–1.34)	1.33 (1.26–1.41)	314	1.19 (1.05–1.35)	1.31 (1.15–1.50)
Personal history of breast cancer	No	6585	Reference	Reference	1247	Reference	Reference
	Yes	435	1.68 (1.52–1.84)	1.39 (1.21–1.60)	82	1.66 (1.32–2.06)	1.60 (1.17–2.18)
Hormone replacement therapy in last 6 months	No	6045	Reference	Reference	1158	Reference	Reference
	Yes	973	1.20 (1.12–1.29)	1.24 (1.16–1.33)	171	1.10 (0.94–1.29)	1.18 (1.00–1.39)
Breast surgery or biopsy for benign conditions	Did not report any	5414	Reference	Reference	1048	Reference	Reference
	Yes	1606	1.37 (1.30–1.45)	1.24 (1.18–1.32)	281	1.24 (1.08–1.41)	1.03 (0.90–1.18)
Self-reported breast symptoms	Did not report any	6527	Reference	Reference	1071	Reference	Reference
	Yes	493	7.65 (6.96–8.39)	7.44 (6.76–8.20)	258	24.0 (20.9–27.4)	22.7 (19.6–26.3)

SES, socio-economic status; IRSD, Index of Relative Socio-economic Disadvantage. * pT2 or above pathological tumor size. Personal history of ovarian cancer (*n* = 21) had univariable OR of 0.57 (95% CI 0.36–0.86); because our models predict increased risk for breast cancer, this co-variate was not included in the multivariable models given the reduced OR in keeping with the ovarian cancer treatment effect on breast cancer risk.

**Table 2:** Univariable and multivariable models to predict interval cancers.

		Interval Cancers		
			Univariable	Multivariable
Characteristic	Category	N events	HR (95% CI)	HR (95% CI)
Age group	40–49	283	Reference	Reference
	50–59	728	0.87 (0.76–1.00)	0.95 (0.81–1.10)
	60–69	625	0.91 (0.79–1.05)	1.09 (0.93–1.28)
	≥70	222	1.14 (0.95–1.35)	1.41 (1.16–1.72)
Postcode-based SES (IRSD) *	1st quintile (most disadvantaged)	145	Reference	Reference
	2nd quintile	426	1.26 (1.04–1.52)	1.20 (0.99–1.46)
	3rd quintile	339	1.16 (0.95–1.41)	1.08 (0.89–1.33)
	4th quintile	283	1.21 (0.99–1.47)	1.11 (0.90–1.36)
	5th quintile (least disadvantaged)	660	1.36 (1.14–1.63)	1.25 (1.04–1.50)
Screen round/time since last screen	First screen	254	0.91 (0.79–1.04)	0.90 (0.78–1.06)
	<15 months	85	0.94 (0.75–1.18)	1.05 (0.82–1.35)
	≥15, <27 months	1112	Reference	Reference
	≥27 months	407	1.12 (1.00–1.25)	0.90 (0.78–1.06)
Breast density	Not dense	572	Reference	Reference
	Dense	404	2.42 (2.20–2.66)	2.36 (2.14–2.61)
First-degree family history of breast cancer	Did not report any	1481	Reference	Reference
	Yes	377	1.22 (1.09–1.37)	1.17 (1.03–1.33)
Hormone replacement therapy in last 6 months	No	1524	Reference	Reference
	Yes	334	1.61 (1.43–1.81)	1.52 (1.34–1.72)
Breast surgery or biopsy for benign conditions	Did not report any	1368	Reference	Reference
	Yes	490	1.70 (1.54–1.89)	1.50 (1.35–1.68)
Self-reported breast symptoms	Did not report any	1787	Reference	Reference
	Yes	71	3.86 (3.05–4.89)	3.27 (2.53–4.24)

SES, socio-economic status; IRSD, Index of Relative Socio-economic Disadvantage. Personal history could not be included in the model for interval cancers because recurrent or second breast cancers are not routinely reported to cancer registries. Personal history of ovarian cancer (*n* = 21) had a univariable HR of 0.57 (95% CI 0.36–0.86); because our models predict increased risk for breast cancer, this co-variate was not included in the multivariable models given the reduced HR in keeping with the ovarian cancer treatment effect on breast cancer risk. * IRSD is a socio-economic index that summarises a range of information about the economic and social conditions of people and households within an area including income, qualifications, and low skilled occupations.

## Data Availability

Subject to approval by the data custodian BreastScreen WA.

## Supplementary Materials

The following supporting information can be downloaded at: https://www.mdpi.com/article/10.3390/cancers17050810/s1, Table S1. Characteristics of screening episodes; Figure S1.1 ROC for screen-detected cancers overall; Figure S1.2 Calibration plot for screen-detected cancers overall; Figure S2.1 ROC for interval cancers; Figure S2.2 Calibration plot for interval cancers.
