# Supplementary material for: Risk Models to Predict Screen-Detected and Interval Breast Cancers in Population Mammography Screening Participants
Source: Cancers (Basel). 2025 Feb 26;17(5):810. doi: 10.3390/cancers17050810 (PMC11898880; doi:10.3390/cancers17050810)
Supplement: Supplementary file 1 [file cancers-17-00810-s001.zip › cancers-3491044-supplementary.pdf]

cancers

MDPI

Table S1. Characteristics of screening episodes.

Characteristics	Category	N (%)
Age	40-49	142700 (13.9%)
	50-59	424213 (41.3%)
	60-69	356073 (34.7%)
	≥70	103151 (10.1%)
Postcode-based SES (IRSD)	1st quintile (most disadvantaged)	98683 (9.6%)
	2nd quintile	231500 (22.6%)
	3rd quintile	199763 (19.5%)
	4th quintile	160137 (15.6%)
	5th quintile (least disadvantaged)	331041 (32.3%)
	Unknown or interstate	5013 (0.5%)
Screen round and time since last screen	First screen	148499 (14.5%)
	<15 months	92484 (9.0%)
	15<=, <27 months	591503 (57.6%)
	>=27 months	193651 (18.9%)
Breast density	Dense	235476 (23.0%)
	Not dense	703213 (68.5%)
	Unknown	87448 (8.5%)
Personal history of breast cancer	Yes	39086 (3.8%)
	No	987049 (96.2%)
	No response	2 (<0.1%)
Personal history of ovarian cancer	Yes	5319 (0.5%)
	No	1020811 (99.5%)
	No response	7 (<0.1%)
First-degree family history of breast cancer	Yes	211742 (20.6%)
	Did not report any	814395 (79.4%)
Hormone replacement therapy in last 6 months	Yes	121189 (11.8%)
	No	904850 (88.2%)
	No response	98 (<0.1%)
Breast surgery or biopsy for benign conditions	Yes	182562 (17.8%)
	Did not report any	843575 (82.2%)
Self-reported breast symptoms	Yes	10455 (1.0%)
	Did not report any	1015682 (99.0%)
BSWA's high risk category*	Yes	145086 (14.1%)
	No	881051 (85.9%)

SES, socio-economic status; IRSD, Index of Relative Socio-economic Disadvantage; BSWA, Breast-Screen Western Australia. *Women with established risk factors listed in the Methods section were invited every year.

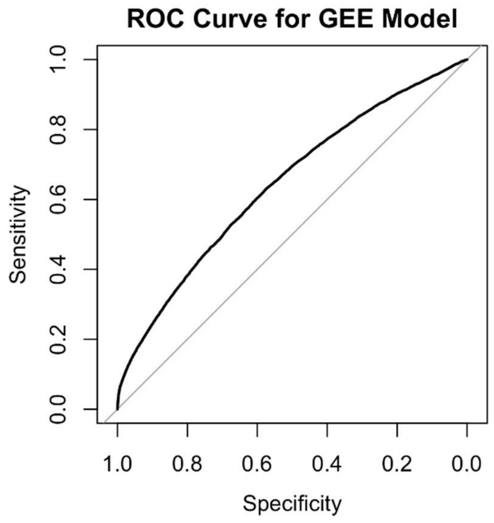

Figure S1.1 ROC for screen-detected cancers overall.

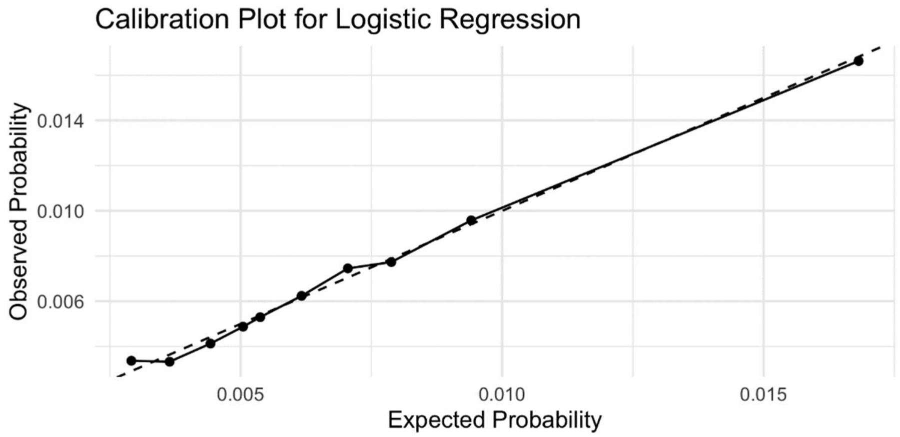

Figure S1.2 Calibration plot for screen-detected cancers overall.

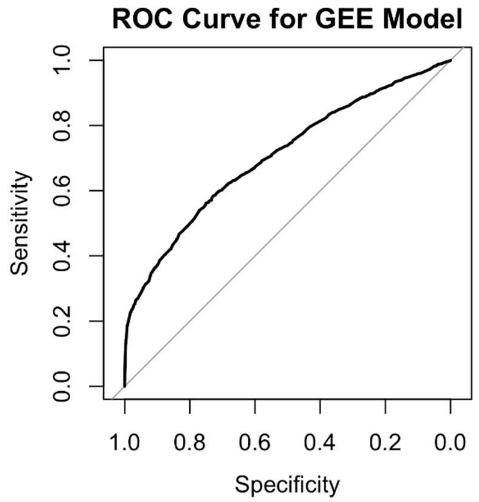

Figure S2.1 ROC for interval cancers.

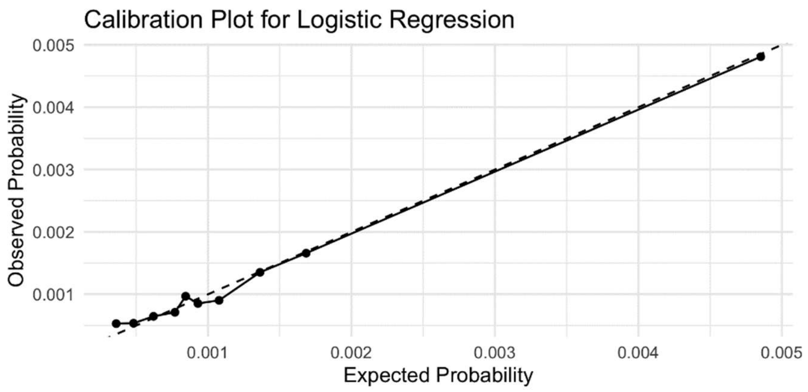

Figure S2.2 Calibration plot for interval cancers.
